# The enemy of my enemy is my friend

**DOI:** 10.1172/JCI183885

**Published:** 2024-07-15

**Authors:** Sarah Voss

**Affiliations:** Johns Hopkins Medical Institute, Baltimore, Maryland, USA.

It is a muggy February morning, 2040, and Myra is ascending the new rock wall at the climbing gym. Her hands strain to hold on as the ledge she is gripping slips between her fingers. Myra falls. In a sore pile on the floor, she thinks, “That’s enough for today,” as she looks at her hands and notices a stream of blood.

The blood is easily washed away, but over the next few days, the cut reddens, swells, and becomes sore. An infection begins to set in. Myra deposits a small sample of the infection into an adaptor on her smartphone and the screen flashes:

SPECIES: *Staphylococcus aureus*

DRUG RESISTANCE PROFILE: Vancomycin, methicillin, daptomycin, linezolid...

TREATMENT: Topical bacteriophage

Relieved, Myra picks up her prescription at the local drug store, administers the bacteriophage, and the infection fades within days.

Thankfully for Myra, she lives in a future that is not dependent on antibiotics to treat bacterial infections. Were someone infected with this strain of *S*. *aureus* today, there would be no available treatments, as this strain is resistant to all currently available antibiotics. Due to its prevalence, antibiotic-resistant *S*. *aureus* caused more than 100,000 deaths in 2019, and antibiotic-resistant strains are present in all major pathogenic bacterial species (1).

Researchers estimated that in 2019, 4.95 million deaths were associated with antibiotic-resistant infections, and that number is predicted to rise to 10 million deaths per year by 2050 (1, 2). In addition to the loss of life, the World Bank predicts that antibiotic-resistant infections will cost the US $1 trillion in additional healthcare costs by 2050 and cause the US to lose $1 to $3.4 trillion in gross domestic product per year by 2030 (3).

So how can we avoid this bleak future? For many years, the solution was new antibiotics. But while it takes decades for researchers to develop a new antibiotic, it historically takes *S*. *aureus* two years to develop resistance to new antibiotics (4). Instead, many scientists hope to develop the bacteria’s natural predator, the bacteriophage (phage), into a solution for this festering crisis.

Phages are viruses that infect and destroy bacterial cells, but not human cells. Because phages are highly specific for individual bacterial strains, they can target pathogenic bacteria without disrupting the natural microbial community of the body, unlike antibiotics. Although phages are not yet clinically approved in the US, they have been used in “compassionate use” cases to cure patients who otherwise would have died of antibiotic-resistant infections (5). While the treatment has the potential to save many lives, there are still several barriers scientists need to address before phage therapy can be widely used, notably, the human immune response to the phage and the presence of bacterial defense systems.

The human immune system is a complex network that can be triggered by the presence of foreign particles. Though phages make up part of the natural human microbial community, some studies have found that antibodies, the immune system’s adaptive, protective particles, are generated in response to phage therapy, implying the body would kill the phage and prevent it from curing the patient (6). However, other studies have shown phages elicit antibody production in some individuals, but not others, and the presence of antibodies was not correlated with treatment outcomes (7). Currently, it is unclear how antibodies impede therapeutic phages, how the immune system identifies phages, and whether phages can be designed or chosen to avoid immune detection.

Just as humans have immune systems that help protect us against microbial invaders, bacteria also have immune systems, termed bacterial defense systems, that help protect them from phage infections. A notable example is the CRISPR/Cas system, which has been adapted as a gene-editing technology, but originally evolved in bacteria to destroy incoming phage DNA. These bacterial defense systems have the potential to prevent successful phage therapy by destroying the phages used for treatment. Fortunately, there has been a recent boom in the discovery of new bacterial defense systems, allowing scientists to identify the barriers preventing a phage from killing a bacterial infection (8). Currently, researchers are working to understand how the newly discovered bacterial defense systems kill phages and, in turn, how phages can be designed to circumvent the bacterial defense systems within pathogenic strains.

Through our looking glass to the future, we can see a now-healthy Myra on her treadmill. She runs as fast as she can, yet stays in the same place. In our ever-evolving world, we also must run and adapt or risk being outcompeted. Though many bacterial infections are currently treatable with antibiotics, we may be at the precipice of an antibiotic-resistant pandemic, and the development of new treatments for bacterial infections, such as phage therapy, are vital for the sustained health and prosperity of our communities.

## Version 1 07/15/2024

Electronic publication
